# Precision Mapping of Amyloid-β Binding Reveals Perisynaptic Localization and Spatially Restricted Plasticity Deficits

**DOI:** 10.1523/ENEURO.0416-21.2021

**Published:** 2021-12-10

**Authors:** Hannah S. Actor-Engel, Samantha L. Schwartz, Kevin C. Crosby, Brooke L. Sinnen, Olga Prikhodko, Harrison J. Ramsay, Jennifer N. Bourne, Christina S. Winborn, Alexandra Lucas, Katharine R. Smith, Mark L. Dell’Acqua, Matthew J. Kennedy

**Affiliations:** ^1^Department of Pharmacology, University of Colorado School of Medicine, Anschutz Medical Campus, Aurora, CO 80045; ^2^Electron Microscopy Core, University of Colorado Anschutz Medical Campus, Aurora, CO 80045

**Keywords:** amyloid-β, LTP, plasticity, postsynaptic density, super-resolution, synapse

## Abstract

Secreted amyloid-β (Aβ) peptide forms neurotoxic oligomeric assemblies thought to cause synaptic deficits associated with Alzheimer’s disease (AD). Soluble Aβ oligomers (Aβo) directly bind to neurons with high affinity and block plasticity mechanisms related to learning and memory, trigger loss of excitatory synapses and eventually cause cell death. While Aβo toxicity has been intensely investigated, it remains unclear precisely where Aβo initially binds to the surface of neurons and whether sites of binding relate to synaptic deficits. Here, we used a combination of live cell, super-resolution and ultrastructural imaging techniques to investigate the kinetics, reversibility and nanoscale location of Aβo binding. Surprisingly, Aβo does not bind directly at the synaptic cleft as previously thought but, instead, forms distinct nanoscale clusters encircling the postsynaptic membrane with a significant fraction also binding presynaptic axon terminals. Synaptic plasticity deficits were observed at Aβo-bound synapses but not closely neighboring Aβo-free synapses. Thus, perisynaptic Aβo binding triggers spatially restricted signaling mechanisms to disrupt synaptic function. These data provide new insight into the earliest steps of Aβo pathology and lay the groundwork for future studies evaluating potential surface receptor(s) and local signaling mechanisms responsible for Aβo binding and synapse dysfunction.

## Significance Statement

Amyloid-β (Aβ) is one of the principal neurotoxic agents responsible for Alzheimer’s disease (AD). Defining where Aβ attaches to neurons is critical for understanding its toxicity and role in disease. Here, we used high resolution microscopy techniques to demonstrate Aβ rapidly forms stable nanoscale clusters immediately adjacent to a subset of excitatory synaptic connections. Synaptic plasticity was only impaired at Aβ-targeted synapses and not at neighboring Aβ-free synapses. Thus, perisynaptic Aβ binding rapidly triggers locally restricted signaling mechanisms underlying its synaptic toxicity.

## Introduction

Amyloid-β (Aβ) is widely recognized as a primary neuropathologic agent in Alzheimer’s disease (AD). Formed by proteolytic processing of amyloid precursor protein (APP), Aβ peptide self-associates to form soluble oligomers and fibrils before eventually depositing into the hallmark plaques associated with AD (Seubert et al., 1992; Shoji et al., 1992; Lambert et al., 1998; Gong et al., 2003; Lesné et al., 2006). While Aβ plaques correlate with neuronal dysfunction and cell death, considerable evidence supports a major role for soluble, oligomeric Aβ (Aβo) in synapse toxicity. For example, synapse loss and memory impairment in AD can occur before widespread plaque formation (Dekosky and Scheff, 1990; Terry et al., 1991). Acute exposure to nano- to picomolar quantities of Aβo, either *in vivo* or *in vitro,* is sufficient to block neural plasticity within minutes, trigger synapse elimination over days, and eventually cause cell death (Walsh et al., 2002; Shankar et al., 2007, 2008; Wei et al., 2010; Sinnen et al., 2016).

Pioneering studies demonstrated Aβo preferentially accumulates at excitatory synapses within minutes following application of synthetic Aβ-derived diffusible ligands (ADDLs; Lacor et al., 2004; Koffie et al., 2009; Renner et al., 2010). Consistent with these studies, naturally derived Aβ produced over much longer timescales (months/years) also accumulates at synaptic sites in both animal AD models and human AD patients (Games et al., 1995; Gong et al., 2003; Koffie et al., 2009; Pickett et al., 2016). While these observations suggest a direct role of Aβ in synapse toxicity, little is known about the earliest steps of Aβo binding. Precisely where does Aβo engage neurons relative to synaptic connections? Does it bind to presynaptic or postsynaptic compartments? How fast does it associate and dissociate? Are only Aβo-bound synapses impaired? Addressing these questions will be important for understanding the mechanisms of Aβo toxicity. For example, mapping where Aβo binds to neurons with nanometer precision will be imperative for evaluating putative Aβo receptors. Thus far, over 20 Aβ receptors have been described, each with diverse subcellular localizations, including the presynaptic membrane, the postsynaptic membrane, perisynaptic and nonsynaptic sites, yet whether Aβo directly binds at these sites remains unclear. Furthermore, whether Aβo binding triggers cell wide synaptic dysfunction or selectively impairs synapses to which it is bound is not known.

Using longitudinal live imaging, we demonstrate acutely applied Aβo rapidly forms stable clusters on the neuronal cell surface. In agreement with previous studies, Aβo preferentially associates with excitatory synapses. However, super-resolution light microscopy, immunogold electron microscopy (EM), and expansion microscopy (ExM) revealed that Aβo does not bind directly at the synaptic cleft, but instead forms stable nanoscale clusters encircling the postsynaptic membrane with a significant fraction also binding the presynaptic axon terminal. Finally, we used two-photon glutamate uncaging at individual synapses to demonstrate plasticity deficits are restricted to Aβo-bound spines. Together, these results provide the first quantitative, super-resolution interrogation of the earliest steps of Aβo binding, dynamics and local toxicity at synaptic sites. Defining precisely where Aβo initially engages the neuronal surface is a key step in understanding how Aβo causes synaptic dysfunction and for directing future strategies aimed at preventing Aβo-induced pathology.

## Materials and Methods

### Cell culture and transfection

All animal procedures were conducted in accordance with the Institutional Animal Care and Use Committee at the University of Colorado, Anschutz Medical Campus. Primary hippocampal cultures were made from postnatal day (P)0 to P1 Sprague Dawley rats as previously described (Sinnen et al., 2016) and maintained in Neurobasal media (Invitrogen) with B27 supplement (Invitrogen) for 15–18 d *in vitro* (DIV) before experiments. Neurons were typically transfected between DIV15 and DIV17 using Lipofectamine 2000 according to the manufacturer’s instructions. Plasmids used in this study include: PSD95_FingR_-GFP (Gift from Don Arnold, University of Southern California) and pCAG-mCh, pCAG-GFP and pSyn-tdtomato plasmids (where pCAG is the chicken β-actin promoter; pSyn is human synapsin promoter).

### Aβ preparation

Soluble Aβ1-42 (Anaspec) oligomers were prepared similar to a previously reported method (Klein, 2002). Briefly, Aβ was dissolved in 1,1,1,3,3,3-hexafluoro-2-propanol, aliquoted and dried in a chemical fume hood and stored at −80. The day before use, Aβ (6 nmol) was dissolved in 4 μl of dimethyl sulfoxide and then 60 μl of PBS was added for a final concentration of 94 μm. The dissolved peptide was incubated at 4°C overnight. Following 12–24 h of incubation, the sample was centrifuged at 14,000 × *g* at 4°C. The supernatant was reserved and applied to a size exclusion spin filter (30-kDa cutoff; Millipore, MRCFOR030) and centrifuged for 10 min at 10,000 × *g* at room temperature to remove low molecular weight Aβ species. The high molecular weight fraction was diluted to a final volume of 600 μl with PBS (working concentration of 10 μm) and stored on ice until use. For experiments using fluorescent Aβo, the preparation was conducted as described above with HiLyte647-conjugated Aβ (AnaSpec) included at a molar ratio of 1:3, labeled:unlabeled Aβ peptide.

### Live-cell imaging

Live-cell imaging of dissociated neurons was performed at 31°C on an Olympus IX71 equipped with a spinning-disk scan head (Yokogawa). Excitation illumination was delivered from an acousto-optic tunable filter (AOTF) controlled laser launch (Andor).

Images were acquired using a 60× Plan Apochromat 1.4 numerical aperture objective and collected on a 1024 × 1024-pixel Andor iXon EM-CCD camera. Data acquisition and analysis were performed with MetaMorph (Molecular Devices), Andor IQ, and ImageJ software.

For live cell Aβo binding experiments, z-stacks were acquired every 10 s. Labeled Aβ was added to the imaging chamber following a baseline acquisition imaging period. For Aβo dissociation experiments, Aβo was added to the imaging chamber and allowed to bind for 10 min. The imaging media was then exchanged by washing 3× with Aβo-free media. Synapse-associated Aβo was quantified by creating a binary mask based on the postsynaptic density protein 95 (PSD95) signal and then calculating the average, background-subtracted integrated density. Extrasynaptic Aβo was quantified within a mask created by subtracting the PSD95 mask from a cell fill mask. Binding kinetics were calculated by fitting plots of the Aβo fluorescent signal (F/F_0_) versus time with a single exponential function.

### Fluorescence recovery after photobleaching (FRAP)

Aβo was added to coverslips for at least 10 min to allow binding to saturate. Baseline images were acquired once every 15 s for 12 frames. Aβo puncta were bleached using galvanometric mirrors (FRAPPA module, Andor Technologies) to steer a diffraction limited excitation spot over the region of interest. Photobleaching was typically conducted using 60% laser power from a fiber-coupled 100-mW 641-nm laser with a dwell time of 1 ms. Following photobleaching, images were acquired at 1 frame/min for 25 min.

### Structural LTP/2-photon glutamate uncaging

Structural long-term potentiation (LTP)/two-photon glutamate uncaging two-photon glutamate uncaging and imaging were conducted using a Bruker Optima laser scanning microscope equipped with a Mai-Tai DeepSee laser (Spectra-Physics) for imaging and a Mai-tai laser (Spectra-Physics) for uncaging. Hippocampal neurons transfected with green fluorescent protein (GFP) or tdTomato expressing plasmids were treated with Aβo generated with either HiLyte568 or HiLyte488-labeled Aβ peptide respectively. Full z-stacks were acquired to identify Aβo-bound spines using sequential 920/1040-nm excitation (GFP/HiLyte568) or single 920-nm excitation (tdTom/HiLyte488 Aβo). Aβo-positive and negative spines were subject to glutamate uncaging in artificial CSF (ACSF) containing 3 mm Ca^2+^ and lacking Mg^2+^. Uncaging power and duration were calibrated so that dendritic spine Ca^2+^ influx triggered by glutamate uncaging (measured in separate cells expressing GCaMP6) matched Ca^2+^ influx resulting from spontaneous glutamate release (Sinnen et al., 2016). Spine growth was triggered by MNI-glutamate (2 mm) uncaging at 720 nm with a train of 45 1-ms pulses delivered at 0.5 Hz at a single spot adjacent to the tip of the targeted spine. A mix of Aβo-positive and negative spines were selected from each cell. Z-stacks were acquired every 90 s to visualize spine morphology preglutamate and postglutamate uncaging.

### Immunocytochemistry

Cultured neurons were fixed with 4% PFA for 10 min at room temperature, permeabilized with 0.1% triton for 10 min, and blocked with 5% BSA in PBS for 30 min. Primary antibodies were diluted with PBS containing 3% BSA and added to fixed cells for 2 h at room temperature. Anti-β-amyloid 1–16 (Biolegend 6E10, catalog #803014; 1:1000), PSD95 (Millipore, catalog #MAB1596; 1:1000), GluA1-Polyclonal (1:300; Kennedy et al., 2010; Hiester et al., 2017), Gephyrin (Synaptic Systems catalog #147011; 1:1000), and Bassoon (Synaptic Systems catalog #141004; 1:1000). Cells were washed with PBS in between primary and secondary incubations. Samples were incubated with fluorescently conjugated secondary antibodies (1:2000) for 1 h at room temperature.

### Structured illumination microscopy (SIM)

Multichannel SIM images of Aβ-treated neurons were acquired with a Nikon N-SIM E SIM using a 100 × 1.49 NA objective, and reconstructed using Nikon Elements software as described previously with minor modifications (Smith et al., 2014; Crosby et al., 2019). Imaging parameters (laser power, exposure) were optimized for a high signal-to-noise ratio (>8). For each coverslip imaged, the objective correction collar was adjusted automatically and a Fourier transform image was used to confirm optimal correction collar adjustment. Z-stacks (z = 0.2 μm, 13 slices) were reconstructed using Nikon Elements software. For three-dimensional (3D) stack reconstruction, the illumination modulation contrast was set automatically and the high-resolution noise suppression was set to 1, and kept consistent across all images.

### SIM analysis

Quantification of Aβ density distribution relative to specific proteins of interest was performed using custom analysis software written in MATLAB along with the freely available MATLAB Toolbox DipImage (Delft). Proteins of interest were identified as follows. Each channel of the image was smoothed using a Laplacian or Gaussian filter to enhance punctate objects with a kernel size of two pixels in *X* and *Y* and one pixel in *Z*. An automatic intensity threshold was calculated using the MATLAB multithresh function to identify two threshold levels based on the image intensity histogram. The higher threshold was used to generate a mask for each image. The DipImage label function was then used to identify individual objects from the mask and then a 3D Euclidean distance transform was applied using the MATLAB function bwdistc1.m, (Mishchenko, 2015), resulting in a new distance image in which each voxel of the image represents the 3D distance to the closest masked object. To mask the Aβ signal a similar process was used, however, because the Aβ puncta were more densely spaced, an additional watershed filter was used to improve segmentation. Watershed lines were computed from the Gaussian filtered (sxy = 1) original image with a connectivity of 1 pixel on a frame-to-frame basis using the DipImage gaussf and watershed functions and then subtracted from the Aβ mask. The center of mass positions for each labeled Aβ puncta were next identified using the DipImage measure function. The distance image could then be used to identify the shell of voxels within a specified distance from the proteins of interest. The count of Aβ puncta center positions within this volume of voxels approximates the Aβ density within the specified distance range. Resulting densities were then divided by a normalization term representing the expected density from a uniform Aβ distribution, such that values >1 represent an Aβ density above uniform. To generate this normalization term, a simulation for each image was performed by randomly distributing the same number of Aβ puncta found in the original image within 642 nm (20 × *X-Y* pixel size) of the proteins of interest and then calculating the density within each distance range. To prevent any artifact arising from the difference in *Z* pixel size compared with *X-Y* pixel size in SIM images, the number of Aβ puncta at each *z* plane was kept the same between the original image and the simulated image.

### Direct stochastic optical reconstruction microscopy (dSTORM) imaging and analysis

Cells exposed to 500 nm Aβo-647 for 10 min or anti-GluA1 for 15 min were fixed and labeled with anti-PSD95 as described above. Secondary antibodies were conjugated to either Alexa Fluor 647 or CF568. Following secondary antibody labeling, cells were postfixed with 4% paraformaldehyde for 15 min. Samples were imaged in a buffer containing 50 mm Cysteamine hydrochloride, 10% glucose, 0.6 mg/ml glucose oxidase from *Aspergillus niger*, 0.063 mg/ml Catalase from Bovine liver in PBS, pH between 7.5 and 8.0. Imaging was performed on a Zeiss Elyra P.1 TIRF microscope using a Zeiss α Plan Apochromat TIRF 100×/1.46 NA oil objective and a tube lens providing an extra factor of 1.6× magnification. Alexa Fluor 647 (or HyLite647) and CF568 dyes were imaged in sequential time-series of ∼20,000 frames each. Image size was 256 × 256 pixels, integration time was 18 ms for both channels. Alexa Fluor 647 or HyLite547 molecules were ground-state depleted and imaged with a 100-mW 642-nm laser at 100% AOTF transmission in ultra-high-power mode (condensed field of illumination), corresponding to ∼1.4 W/cm^2^. Emission light passed through a LP655 filter. CF-568 molecules were ground-state depleted and imaged with a 200-mW 561-nm laser at 100% AOTF transmission in ultra-high power mode, corresponding to ∼2.5W/cm^2^. Emission light was passed through a BP 570–650 + LP 750 filter. For each dye, ground-state return was elicited by continuous illumination with a 50-mW 405-nm laser at 0.01–0.1% AOTF transmission. Excitation light was filtered by a 405/488/561/642 filter placed in front of the camera. Images were recorded with an Andor iXon+ 897 EMCCD. The camera EM gain was set to 100, which yields an effective conversion of 1 photograph electron into 1.65 digital units. The image pixel size was 100 nm *xy*.

### Processing

Raw data were processed through a custom pipeline written in MATLAB (MathWorks) made up of a number of modular elements, described below. The Bio-Formats MATLAB toolbox (Linkert et al., 2010) was used to read Zeiss raw data files into MATLAB. Image data were transferred between MATLAB and FIJI using MIJI (http://bigwww.epfl.ch/sage/soft/mij/). If necessary, raw data were preprocessed with a temporal filter (Hoogendoorn et al., 2014) to remove nonhomogeneous background. The filter radius was set at 51 frames, with a key frame distance of 10 (filter is explicitly calculated only for every 10 frames and interpolated between), the quantile for the filtering was set a 20%. Localization of dye emitters was performed using the ThunderSTORM ImageJ plugin (Ovesný et al., 2014). The camera EM gain was set to 100, which resulted in a photon-to-ADU of 1.65. When the temporal median filter was used, the Offset was set to zero. Image filtering was done with the Wavelet filter setting, with a B-Spline order of three and scale of 2.0. A first pass approximate localization of molecules was achieved with by finding local maximum with a peak intensity threshold of 2.5*std(Wave.F1) and 8-neighborhood connectivity. Weighted least squares fitting of the PSF to achieve subpixel localizations was achieved by use of an integrated Gaussian with a fitting radius of four pixels and an initial σ of 1.5. Localizations were filtered based on the attributes of uncertainty (<20 nm) and σ (100–200 nm for CF568 and 90–190 nm for Alexa Fluor 647 and Hylite-647). Before each experiment a calibration was calculated to correct for shifts and distortions between the acquired fluorescent channels. Subdiffractive beads, labeled with fluorophores in both channels were imaged. The bead positions were fitted and registered between the fluorescent channels. Registered localizations from multiple bead images were compiled into one data-set. Calibration matrices of the shift in *x* and *y* direction between the imaging channels across the full field of view were calculated by either applying a 2D polynomial fit or a localized weighted averaging to the registered bead localizations. In the raw data, the shift and distortion between the imaging channels was up to 100 nm. Applying the calibration to the STORM data yields an RMS error of <15 nm for the channel misalignment. Drift correction was performed using the redundant cross-correlation method described previously (Wang et al., 2014). The segmentation parameter was set at 500 frames, the bin size used in the cross-correlation was 10 nm, and the error threshold for the recalculation of the drift was five pixels.

### dSTORM analysis

Coordinate analysis of our dSTORM data are conceptually similar to methods previously used to classify nanoscale organization at the excitatory synapse (Tang et al., 2016). Synapses for downstream analysis were selected manually from a composite rendered image and ROI coordinates were recorded using a custom ImageJ macro. ROI details were imported into MATLAB using the ReadImageJROI function (https://github.com/DylanMuir/ReadImageJROI). The postsynaptic density and synaptic Aβ localization were segmented using a coordinate-by-coordinate density calculation. Because labeling density could vary greatly, the thresholding parameter was determined from the overall density range of the ROI. Localizations with a local-density in the lower 10% of that range were considered to be outside of the synaptic region/clusters. Boundaries for these regions were delineated using MATLAB’s alphaShape function, with an α value of 100. Only regions with an area of 1.5e3 nm^2^ or greater were considered for analysis. MATLAB’s inShape function was used to determine what percentage of Aβ or receptor localizations fell within the PSD boundary. Aβ and receptor nanoclusters were defined by a cutoff determined by randomizing the experimental localizations assuming a uniform distribution across the synaptic region. The local density threshold for an experimental coordinate to be considered as part of a nano-cluster was set at the mean local density of the randomized dataset plus 2 SDs. The geometric boundaries of individual nano-clusters were again delineated using the alphaShape function, with an α value of 11. Aβ or receptor nanoclusters were classified as overlapping with the PSD if the overlap area had a fraction of 0.23 or greater of the total nanocluster area. The weighted center (mean of coordinates) of each nanocluster was calculated and the center to PSD edge was determined using the nearestNeighbor function. This distance was assigned as zero for PSD overlapping nanoclusters.

### Code/software

All code used for super-resolution image analysis is available at the following links: STORM, https://github.com/VVvanL/structure_SMLM_analysisFile_fromSRPipeline_output; SIM, https://github.com/samanthalschwartz/NeuronAnalysisToolbox.

### Electrophysiology

Field recordings were performed as previously described using two- to three-week-old C57BL/6 mice (Freund et al., 2016). Mice were killed and brains rapidly removed and immersed in ice-cold sucrose-containing cutting buffer (2 mm KCl, 12 mm MgCl_2_, 0.2 mm CaCl_2_, 1.3 mm NaH_2_PO_4_, 10 mm d-glucose, 220 mm sucrose, 26 mm NaHCO_3_, 1.77 mm sodium ascorbate, and 2 mm *N*-acetylcysteine). Coronal slices containing hippocampus (400-μm thickness) were prepared using a McIlwain tissue chopper/slicer and recovered at 27°C for >60 min in ACSF (84.3 mm NaCl, 3 mm KCl, 1.8 mm CaCl_2_, 1.3 mm NaH_2_PO_4_, 4.7 mm MgSO_4_, 26 mm NaHCO_3_, 10 mm glucose, 70.4 mm sucrose, 1.2 mm sodium ascorbate, and 0.65 mm *N*-acetylcysteine). After recovery, a single slice was transferred to a recording chamber and superfused with ACSF at a flow rate of 2–3 ml/min at 31°C. The ACSF contained the following: 124 mm NaCl, 3.5 mm KCl, 1.3 mm MgCl_2_, 2.5 mm CaCl_2_, 1.3 mm NaH_2_PO_4_, 10 mm d-glucose, and mm 26 NaHCO_3_. Field recordings were made with a glass micropipette filled with ACSF placed in CA1 stratum radiatum ∼200–300 μm from the cell body layer. Synaptic field EPSPs (fEPSPs) were evoked with bipolar tungsten electrodes placed in the Schaffer collateral axon pathway. For each slice, an input–output curve was generated by increasing the stimulus voltage and recording the synaptic response until either a maximum was reached or evidence of a population spike was observed on the fEPSP response. The control stimulus intensity was set to 40% to 50% of the maximum synaptic response, and a baseline recording was obtained delivering one test pulse every 20 s for 20 min. To elicit LTP, we delivered two trains of 100-Hz stimuli lasting 1 s each, with an intertrain interval of 5 min. This protocol reliably produced LTP that persisted for >45 min. We recorded the maximum amplitude of the fEPSPs as well as their initial slopes, measured between 10% and 40% from the point of negative deflection.

### EM and immunogold labeling

For EM and immunogold labeling of neuronal cultures, we used osmium-free processing as previously described (Phend et al., 1995). Briefly, samples were fixed with 2.5% glutaraldehyde in 0.1 m phosphate buffer, and sequentially treated with 1% tannic acid (EM Sciences), 1% uranyl acetate, 1% PPD, 0.2% iridium tetrabromide, and then were dehydrated and embedded for sectioning. Following ultrathin microtome sectioning, samples were stained for antibodies against Aβ using previously described postembed gold methods (Aoki, 2003). Briefly, grids, were blocked in tris-buffered saline (0.9% sodium chloride) containing 0.1% Triton X-100 (TBST; pH 7.6) and incubated in primary antibody diluted in TBST at room temperature overnight. The following day, grids were washed with TBST and then blocked with TBST, pH 8.2. Grids were incubated for 1 h in donkey anti-mouse secondary conjugated to 10 nm gold (EMS, 25825). Grids were washed, with TBST, H_2_O, postfixed with 1% glutaraldehyde, and counterstained with Reynold’s lead citrate.

### ExM

Samples were prepared according to the ExM protocol outlined in Zhang et al. (2020). Live DIV16 hippocampal cultures were incubated with rabbit anti-GluA1 for 10 min before fixation (Kennedy et al., 2010; Hiester et al., 2017) Aβo (500 nm) was added to live cells for 15 min at 37°C. Cells were fixed for 15 min using 4% paraformaldehyde (Electron Microscopy Sciences) and washed with PBS. Cells were blocked for 30 min in PBS containing 5% bovine serum albumin (Sigma) and stained with mouse anti-Aβ (6E10, Biolegend, 803014). Cells were then permeabilized with 0.2% Triton X-100 (Fisher Scientific) and incubated with guinea pig anti-bassoon (Synaptic Systems, 141-004). Following incubation with secondary antibodies (secondary antibodies were generated in goat and include anti-rabbit Alexa Fluor 488; anti-rabbit Alexa Fluor 568; anti-guinea pig Alexa Fluor 488; anti-guinea pig Dylight 550; anti-mouse Abberior Star 635) cells were exposed to a second round of fixation with 3% PFA/0.1% glutaraldehyde (Electron Microscopy Sciences) and sequentially washed in 1× PBS and distilled water. Acroloyl-X SE (AcX; Invitrogen) diluted at 1:100 in PBS was added to each well and cells were refrigerated overnight. On day 2 of the protocol, the AcX solution was removed and coverslips were washed twice in 1× PBS for 5 min (samples were placed on ice at the start of the second wash). Gelation solution was prepared by combining chilled reagents: Stock X (Zhang et al., 2020), TEMED (Fisher Bioreagents), and ammonium persulfate (APS; Sigma) at a volumetric ratio of 98:1:1. After removing PBS, 500 μl of gelation solution was added to each well for 5 min on ice. Coverslips were then placed cell-side down atop a gelation chamber constructed using a glass microscope slide and cover glass (Zhang et al., 2020); 60 μl of gelation solution was quickly added underneath each coverslip, and the chambers incubated at 37° for 1 h in a humidified chamber. Gelled samples were submerged in 10-ml digestion buffer (Zhang et al., 2020) containing proteinase K in a 100 × 20-mm Petri dish (Corning) on an orbital shaker at 60 rpm at room temperature overnight. On day 3 of the protocol, digestion buffer was removed and gels were incubated with 10-ml distilled water on an orbital shaker at 60 RPM for 10 min to allow for gel expansion. This was repeated two additional times. The expansion process was then repeated a third time, for 20 min. Following the final expansion step, gels were cut and plated on 35 × 10-mm glass-bottom plates (Ted Pella) coated with 0.1% poly-L-lysine (Sigma). A total of 2 ml of distilled water was then added to each plate and samples imaged on a spinning disk confocal microscope.

### ExM analysis

Aβ distribution along the presynaptic to postsynaptic axis was calculated using custom analysis software in MATLAB. Presynaptic and postsynaptic proteins and Aβ puncta objects were identified, labeled and the center of mass position was calculated as described in the SIM analysis section in Materials and Methods. Synapses were filtered based on the following criteria: (1) they contained both a presynaptic and postsynaptic object, (2) there was an Aβ puncta within 20 pixels in *X-Y* and three pixels in the *Z* dimension, and then further selected manually based on the criterion that the synaptic alignment relative to the imaging plane resulted in good separation between presynaptic and postsynaptic objects. Regions were selected in ImageJ and imported into MATLAB using the function ReadImageJROI.m Dylan Muir 2014. For each synapse, the center position for all Aβ puncta with 20 pixels in *X-Y* and three pixels in *Z* was mapped onto a cylindrical coordinate system having a longitudinal axis defined by the vector between the postsynaptic and presynaptic center of mass positions with an origin at the midway point.

### Figure processing

In some cases, images were expanded 2× and were interpolated for display only. Volume-rendered images were created using expanded, masked images through the ImageJ volume viewer with a *z*-aspect of 2. All quantitative analysis was performed on raw image files.

### Statistical analysis

Statistical significance for experiments comparing two populations was determined using a two-tailed unpaired Student’s *t* test. When populations were not normally distributed, Mann–Whitney tests were used. In the cases where the two populations represented paired measurements, a paired Student’s *t* test was used. For experiments comparing three or more populations, a One-way ANOVA with Bonferroni multiple comparison test was used. When populations were not normally distributed, Kruskal–Wallis with Dunn’s multiple comparisons test were used. Statistical analyses were performed using GraphPad Prism and Microsoft excel. All data are presented as mean ± SEM unless otherwise stated.

## Results

### Aβo rapidly and stably accumulates on the cell surface at or near excitatory synapses

To directly visualize Aβo as it binds to neurons, we generated fluorescently labeled Aβo using HiLyte647-labeled Aβ peptide as previously described (Lacor et al., 2004; Sinnen et al., 2016). We confirmed the labeled peptide formed oligomeric species by western blotting and that it blocked LTP following high frequency stimulation in acute mouse hippocampal slices (Extended Data Fig. 1-1).

**Figure 1.**
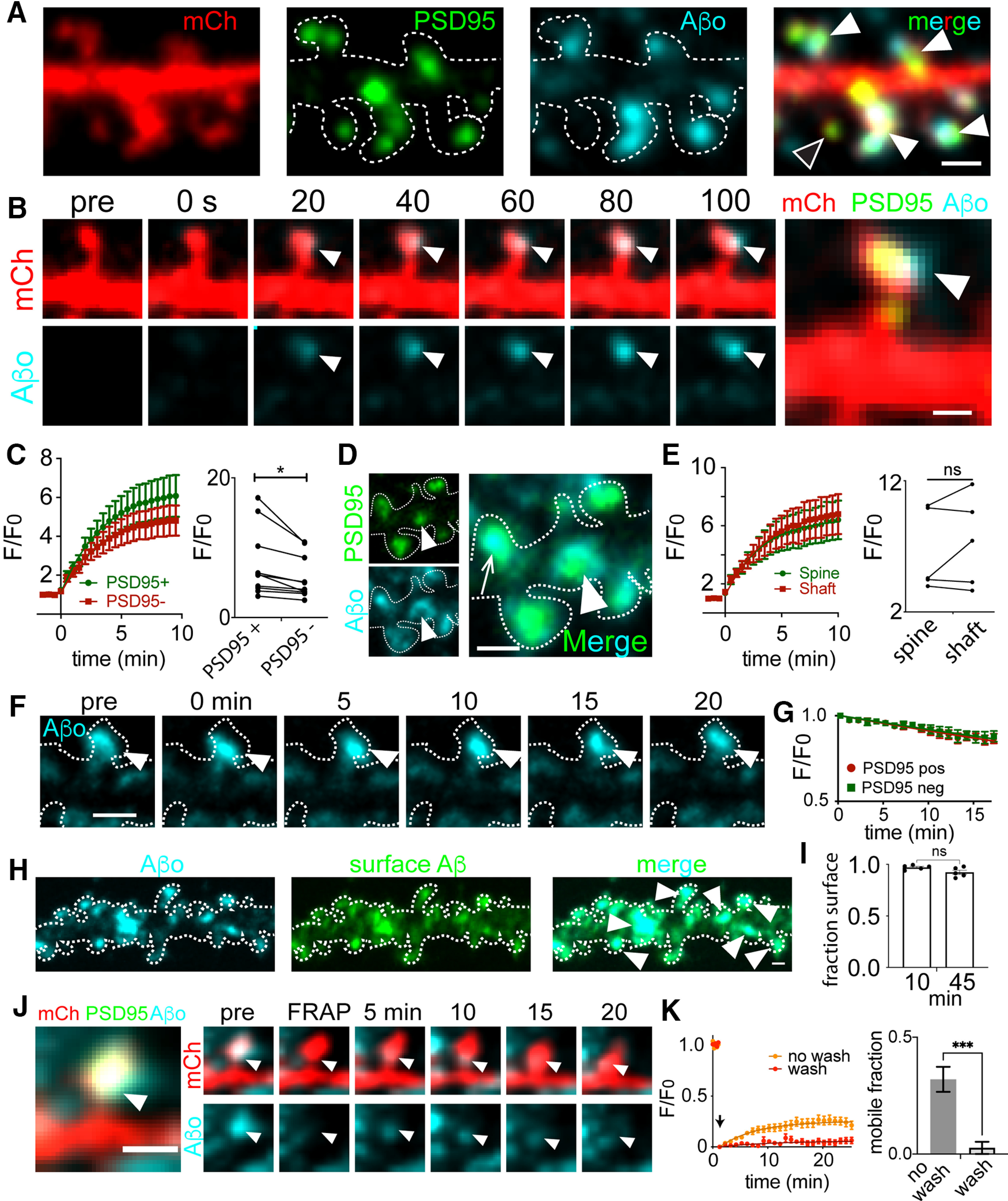
Kinetics of Aβ association and dissociation at synaptic sites. ***A***, Representative dendritic segment from a neuron transfected with mCherry (red) and PSD95_FingR_-GFP (green) treated with labeled Aβo (teal) for 10 min. Closed arrowheads denote dendritic spines labeled with Aβo and the open arrowhead denotes a spine lacking Aβo. Fluorescent Aβ was confirmed to form oligomeric species and block LTP in Extended Data Figure 1-1. Scale bar: 1 μm. ***B***, Representative image series of Aβo accumulation on a spine excitatory synapse. Aβo was added at 0 s. Scale bar: 1 μm. ***C***, left, Quantification of Aβo association kinetics at PSD95-positive dendritic spines or neighboring PSD95-negative dendritic shafts (*n* = 10 neurons, 4 independent cultures). Right, Plateau intensity values for Aβo binding at PSD95+ versus PSD95– (shaft) locations on the same cell (*n* = 10 neurons; *p* = 0.0139, paired Student’s *t* test). ***D***, Representative image of Aβo localization to spine (arrow) and shaft (arrowhead) PSD95. Scale bar: 1 μm. ***E***, left, Aβo binding kinetics at PSD95 puncta on spines or shafts (*n* = 20 PSD95 puncta from 5 neurons, 3 independent cultures). Right, Plateau intensity values of Aβo at PSD95 puncta on the spine and shaft of the same neuron (*n* = 5 neurons, *p* = 0.2857, paired Student’s *t* test). ns = not significant. ***F***, Representative image sequence of Aβo (teal) bound to a dendritic spine following washout into Aβo-free imaging media at *t* = 0 min. The cell outline is shown as a dashed line, drawn based on a cell fill (data not shown). Scale bar: 1 μm. ***G***, Quantification of Aβo intensity following washout. Data are plotted as F/F_0_, with F_0_ representing normalized Aβo signal immediately before washout (*n* = 5 neurons). ***H***, Representative image of a dendrite from a hippocampal neuron expressing mCh (dotted line), treated with labeled Aβo (cyan, left panel) for 10 min and then an extracellular antibody against Aβo to assess surface localization (green). Scale bar: 1 μm. ***I***, Fraction of Aβo puncta (averaged per cell) labeled with an extracellular antibody (i.e., localized to the cell surface) 10 and 45 min following Aβo application (*n* = 5 neurons per group, two independent cultures). ns = not significant, Student’s *t* test. ***J***, Representative time series for FRAP experiments. Shown is a single Aβo-bound spine. The Aβo signal was photobleached and signal recovery was monitored over time. Arrowheads indicate the location of photobleaching and signal recovery. Scale bar: 1 μm. ***K***, Kinetics and extent of Aβo recovery following photobleaching in the continued presence (no wash, orange) or absence (wash, red) of Aβo in the extracellular solution (wash: *n* = 23 spines, from 6 neurons and 2 independent cultures; no wash: *n* = 12 spines from 4 neurons and 2 independent cultures). Quantification of the mobile Aβo fraction is shown to the right under each condition (***p = 0.0007, Student’s *t* test).

10.1523/ENEURO.0416-21.2021.f1-1 Extended Data Figure 1-1 Fluorescent Aβ peptide forms oligomers that disrupt LTP. ***A***, HiLyte647-conjugated Aβ peptide forms oligomeric species. Shown is an immunoblot (probed with anti-Aβ 6E10) of Aβo prepared with HiLyte647-labeled peptide. Note the presence of putative dimers (1), trimers (2), and higher molecular weight species (3) in the preparation. ***B***, Aβo prepared with HiLyte647-labeled peptide disrupts LTP measured by EPSP slope (left) or peak EPSP amplitude (right). LTP was induced by delivering 2 × 1-s trains of 100-Hz stimulation spaced 5 min apart (arrows). Slices were exposed to Aβo for 20 min prior to LTP induction. *n* = 8 slices from 8 animals, control; 6 slices from 6 animals Aβo treated. ***C***, Representative EPSPs from control (left) or Aβo-treated (right) slices before (black) and 45 min following (gray) LTP induction. ***D***, Average EPSP slope (left) and amplitude (right) for control (PBS treated) or Aβo-treated slices 45 min following LTP induction. The dashed line represents baseline; *p < 0.05, Student’s *t* test. Download Figure 1-1, TIF file.

We next characterized the rate of Aβo binding and its stability at different subcellular locations on excitatory neurons. Here, we performed time lapse confocal imaging as we applied labeled Aβo (500 nm) to live dissociated hippocampal neurons expressing mCherry (mCh) to visualize cellular morphology and a GFP-labeled fibronectin intrabody generated with mRNA display (FingR) against PSD95 (PSD95_FingR_-GFP) to label excitatory synapses (Fig. 1*A*). Aβo binding was initially detected as diffraction-limited puncta within seconds of Aβo application. In most cases, Aβ puncta progressively grew in size and intensity but saturated at a maximum plateau value within 5–10 min (Fig. 1*B*,*C*). Once maximum binding was achieved, we observed detectable Aβo signal overlapping with 64 ± 6.8% of excitatory synapses, in agreement with previous studies (Lacor et al., 2004; Sinnen et al., 2016). Aβo binding was not exclusive to synapses; of the total Aβo signal, 50 ± 6.5% associated with dendritic spines and 50 ± 6.5% associated with nonsynaptic sites on the dendritic shaft. Kinetic values for Aβo accumulation at PSD95-positive dendritic spines and nonsynaptic sites were estimated by fitting binding curves with a single exponential function (Fig. 1*C*); τ values for Aβo association with dendritic spines versus nonsynaptic regions on dendritic shafts were similar; 3.62 ± 0.47 and 3.51 ± 0.46 min, respectively. However, when we compared maximum Aβo fluorescence intensity at PSD95-positive dendritic spines (10 min following Aβo addition) with nearby nonsynaptic signal on the dendritic shaft, Aβo signal was consistently elevated near PSD95, suggesting a surface receptor(s) that is enriched at but not exclusive to excitatory synapses (Fig. 1*C*). A possible explanation for this enrichment is that Aβo preferentially associates with a structural feature unique to dendritic spines. Previous studies proposed Aβo binds directly to spine membranes by recognizing their degree of curvature (Sugiura et al., 2015; Terakawa et al., 2018). Contrary to this hypothesis, we found Aβo accumulation at shaft excitatory synapses was not significantly different from spine synapses (Fig. 1*D*,*E*). Thus, Aβo appears to recognize a feature enriched at excitatory synapses regardless of their localization or morphology.

We next measured the stability of Aβo at synaptic and nonsynaptic sites. Here, we added Aβo to hippocampal neurons, allowed binding to saturate for ∼10 min and then washed the cells into Aβo-free extracellular solution (Fig. 1*F*). Following washout, Aβo clusters remained highly stable at both synaptic and nonsynaptic sites with only 13.2 ± 2.8% (synaptic) or 12.7 ± 3.3% (nonsynaptic) loss in signal after 15 min (Fig. 1*G*). We also imaged samples that had been treated with Aβo and then fixed to assess the level of photobleaching over the same imaging time window and found photobleaching could account for 2.2 ± 1.1% loss in signal. We also tested whether Aβo remained on the surface over the time course of this experiment by briefly applying an extracellular Aβ antibody either 10 or 45 min following Aβo application. In both cases we observed nearly all (92.27 ± 0.047% at 45 min) Aβo puncta observed directly with HiLyte647-labeled Aβ peptide colabeled with the antibody signal. Thus, the observed stability was not the result of internalization into stationary intracellular organelles (Fig. 1*H*,*I*).

In a complementary set of experiments, we performed FRAP to investigate surface-bound Aβo dynamics. Here, we applied Aβo, waited 10 min to allow binding to saturate and then focally photobleached individual Aβo puncta at synaptic sites. When we performed FRAP measurements with soluble Aβo (500 nm) remaining in the ACSF, we observed limited but significant recovery of bleached surface Aβo clusters (synaptic Aβo: mobile fraction = 0.25 ± 0.02, recovery rate = 0.15 ± 0.03 min). Under these conditions, the source of signal recovery could be soluble Aβo from the extracellular solution, or laterally diffusing Aβo that was already associated with the cell surface (Renner et al., 2010).To distinguish these possibilities, we performed additional FRAP measurements without Aβo in the extracellular solution to eliminate the soluble pool. Under these conditions, we observed a ∼4-fold decrease in the mobile fraction of bound Aβo (0.059 ± 0.015) at synaptic sites. Thus, while surface-bound Aβo appears stable at steady state, limited exchange with soluble pools can occur (Fig. 1*J*,*K*).

### Super-resolution microscopy reveals Aβo binds predominantly at perisynaptic sites

**Figure 2.**
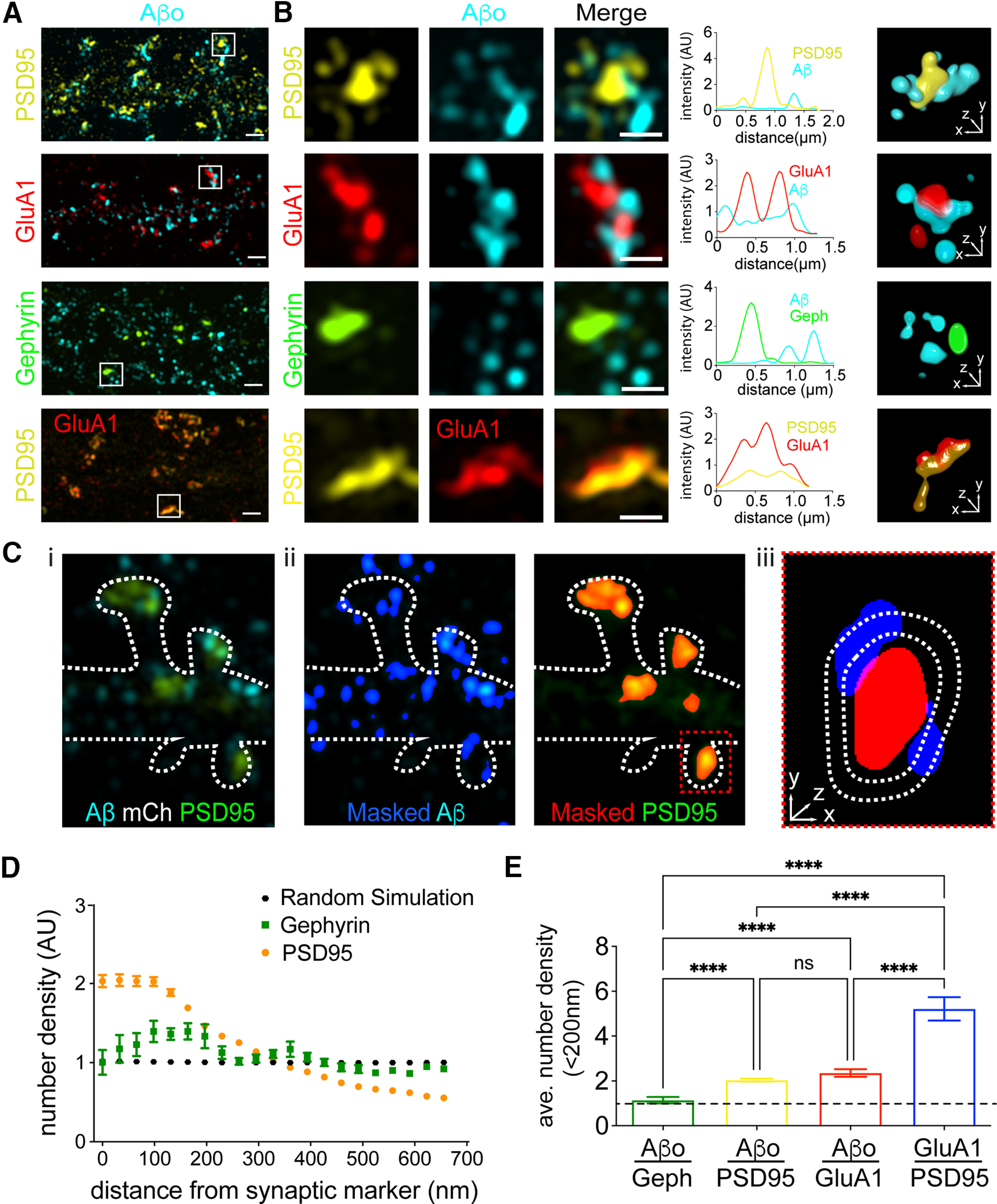
Super-resolution localization of Aβo relative to excitatory and inhibitory synapses. ***A***, Representative SIM images of Aβo (teal) with excitatory synaptic proteins PSD95 (yellow), GluA1 (red), and the inhibitory synaptic protein Gephyrin (green). Bottom panels show control, GluA1 (red) with PSD95 (yellow). Scale bars: 1 μm. ***B***, Expanded regions showing individual synapses from panel ***A***. Scale bars: 500 nm. The graphs to the right of each example plot pixel intensities for each channel along a line drawn diagonally through representative synapses. 3D volume renderings of masked and segmented synapses are shown to the right. ***C***, Approach for quantifying the spatial relationship between synaptic proteins and Aβo. ***i***, Representative SIM image showing PSD95 (green) and Aβo (cyan). The outline of the cell (dashed line) was drawn using the signal from an mCh cell fill (data not shown). ***ii***, The Aβo signal (cyan) is masked and binarized (blue). Right, PSD95 (green) is masked and binarized (red). ***iii***, Magnified red box from ***ii***. The number of Aβo puncta are counted at increasing concentric voxel distances around masked synaptic marker. ***D***, The number of Aβo puncta (quantified as described in panel ***B***) at different distances from either PSD95 (orange; *n* = 48 neurons) or gephyrin (green; *n* = 18 neurons), normalized to randomly localized simulated data (black; average of seven independent simulations). A value of 1 indicates no spatial relationship, >1 a positively correlated spatial relationship, and <1 a negatively correlated spatial relationship. ***E***, Aβo is enriched near the excitatory PSD. Plotted is the average number density of segmented Aβo puncta 0–64 nm from PSD95, GluA1, or gephyrin (PSD95 *n* = 48 neurons, GluA1 *n* = 16 neurons, gephyrin *n* = 18 neurons). The average number density of the synaptic protein GluA1 relative to PSD95 is plotted for comparison (*n* = 7 neurons); ****p < 0.0001, one-way ANOVA. ns = not significant.

The nanoscale distribution of Aβo surface binding remains poorly characterized. We first used SIM (Gustafsson, 2005; Crosby et al., 2019), which has ∼2-fold higher resolution compared with confocal microscopy as well as improved resolution in *z*, to localize Aβo (500 nm, applied 10 min before fixation) relative to the excitatory postsynaptic proteins GluA1 and PSD95, detected by antibody staining (Fig. 2*A*). Surprisingly, our SIM images revealed that most Aβo surface clusters do not actually overlap with PSD95 or GluA1 signal as previously reported in studies using wide field or confocal microscopy (Lacor et al., 2004; Renner et al., 2010). Instead, most Aβo signal appeared immediately adjacent to the PSD (Fig. 2*B*). In contrast, the synaptic receptor GluA1 appeared highly colocalized with PSD95, confirming the observed perisynaptic localization of Aβo was not because of optical or SIM reconstruction artifacts (Fig. 2*B*). Using our SIM dataset, we also wanted to quantify the degree to which total surface-bound Aβo is enriched near excitatory synapses. We developed an unbiased analysis routine that calculates the number of segmented Aβo clusters encountered at different voxel distances from a segmented synaptic marker protein (Fig. 2*C*; for details, see Materials and Methods). The number of Aβo clusters at each distance is then divided by a randomly simulated data set with the same labeling density. Thus, if Aβo binding is random with respect to the protein of interest, our analysis reports a value of one. While there was little direct overlap between Aβo and excitatory synaptic proteins, we did observe a high degree of enrichment of total Aβo signal within 200 nm of excitatory synapses. As a control, we found no significant enrichment of Aβo at inhibitory synapses labeled with gephyrin, confirming specificity for excitatory synaptic connections (Fig. 2*D*; Lacor et al., 2004; Renner et al., 2010). Consistent with perisynaptic or extrasynaptic localization of bound Aβo, our analyses yielded a much greater degree of enrichment of the synaptic protein GluA1 near PSD95 (Fig. 2*E*).

**Figure 3.**
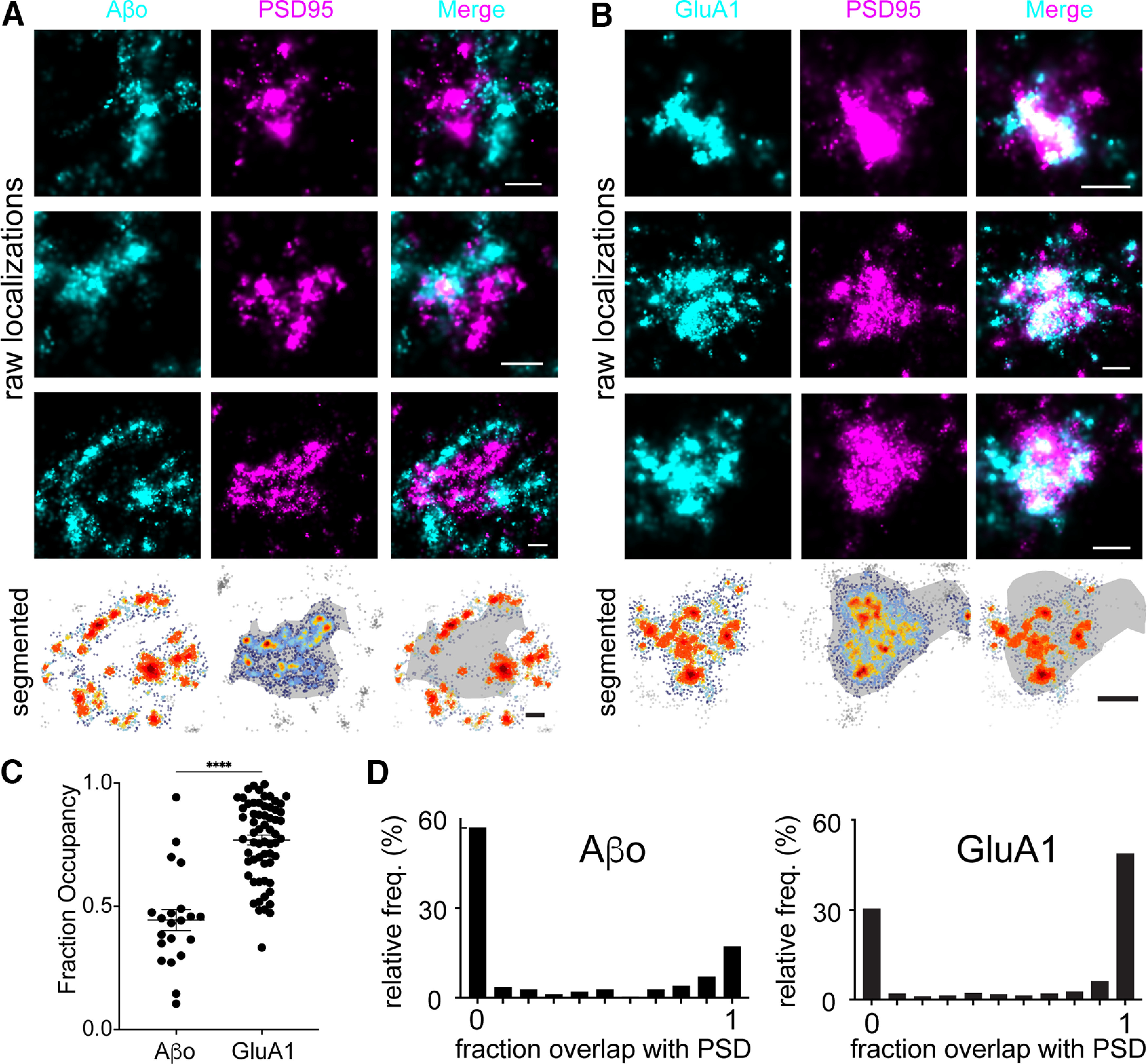
Single molecule localization microscopy reveals Aβo forms nanoscale clusters immediately adjacent to the synaptic membrane. ***A***, Representative dSTORM images of synapses from hippocampal neurons labeled with PSD95 (magenta) that were treated with 500 nm Aβo (cyan) for 10 min. Three examples of raw localization data are shown. The bottom panels display localizations using a density-based lookup table with warmer colors representing higher density regions. The segmented PSD is shaded in gray. Scale bar: 200 nm. ***B***, Representative dSTORM image of GluA1 (cyan) and PSD95 (magenta). Raw localizations are shown in the top panels and the bottom panels are rendered as in ***A***. Scale bar: 200 nm. ***C***, Quantification of the fraction of individual spine Aβo localizations that fall within the segmented PSD95 at single synapses (left; *n* = 24 synapses, 5 cells, 2 independent cultures). The same analysis is shown for GluA1 for comparison (right; *n* = 64 synapses, 11 cells, 3 independent cultures); ***p < 0.0001, Student’s *t* test. ***D***, Frequency histograms are shown plotting the fraction overlap of segmented Aβo clusters (left) or GluA1 clusters (right) with the PSD. A value of 0 indicates no overlap with the PSD while a value of 1 indicates the cluster fell entirely within the PSD. Intermediate values indicate clusters that fell on the edge of the segmented PSD.

To more precisely characterize the nanoscale organization of Aβo binding with respect to synapses, we used dSTORM, which has 3- to 4-fold greater resolution than SIM (Heilemann et al., 2008). Here, we resolved Aβo signal into discrete nanoscale clusters immediately adjacent to, but generally nonoverlapping with the postsynaptic density, labeled by immunostaining PSD95 (Fig. 3*A*). On average, Aβo-bound synapses were associated with 8.04 ± 1.36 Aβo clusters, with individual clusters having an average area of 4548 ± 600 nm^2^. To quantify the degree of Aβo overlap with the PSD, we employed a density-based clustering algorithm to define the PSD (based on a threshold density of PSD95 localizations) and then calculated the fraction of individual Aβo localizations that fell within the segmented PSD region (Fig. 3*A*). For comparison to a known synaptic protein, we also performed this analysis with GluA1 (Fig. 3*B*). While 77 ± 1.9% of GluA1 localizations were observed within the PSD boundary, only 41 ± 4.2% of Aβo localizations fell within the PSD (Fig. 3*C*). We also performed a separate analysis where we segmented discrete, spine-localized Aβo clusters and quantified their percentage overlap with the segmented PSD. Only 12.9% of spine Aβo clusters fully overlapped with the PSD compared with 50.9% for GluA1. Conversely, we observed that 61.1% of spine Aβo clusters were completely excluded from the PSD compared with only 28.7% for GluA1 (Fig. 3*D*). Combined, our super-resolution imaging data reveal the majority of spine-localized Aβo does not actually bind directly at the synaptic cleft, but localizes to perisynaptic sites immediately adjacent to the PSD on the dendritic spine.

### Aβo binds both presynaptic and postsynaptic membranes

**Figure 4.**
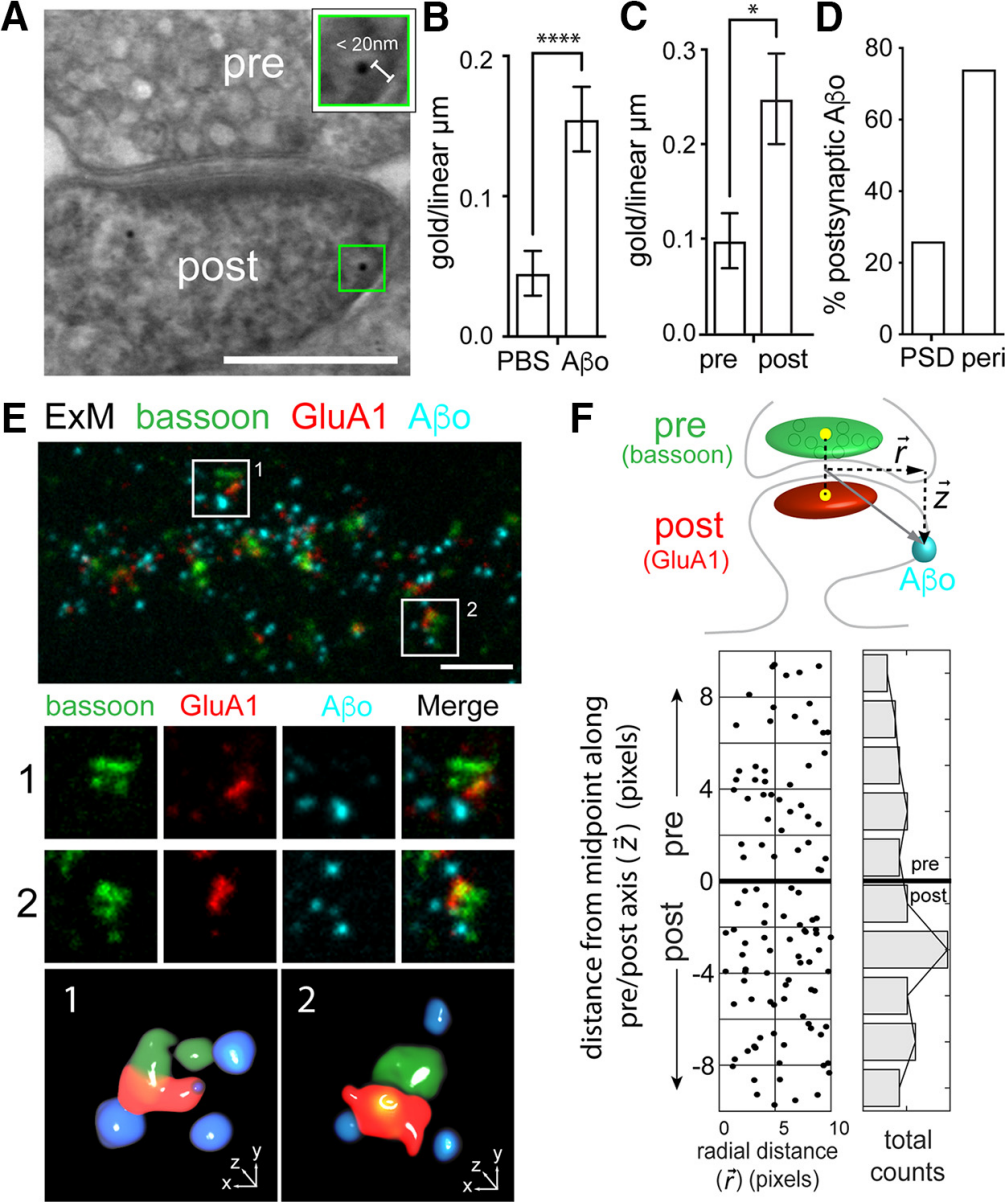
Aβo binds at both presynaptic and postsynaptic sites. ***A***, Representative postembedding immunogold electron micrograph of a synapse exposed to 500 nm Aβo and labeled with an Aβ antibody and gold-conjugated secondary. The green box (magnified in inset) highlights immunogold signal. Gold particles within 20 nm of the cell membrane were considered plasma membrane-associated based on the size of the primary and secondary labeling antibodies. Scale bar: 200 nm. ***B***, Quantification of total membrane-associated Aβo (measured as total number of gold particles per linear micron of plasma membrane) from samples treated with PBS alone or PBS with 500 nm Aβo. (PBS: *n* = 60 spines; Aβ: *n* = 66 spines; *p* < 0.0001, Mann–Whitney test). ***C***, Quantification of Aβo label on the presynaptic or postsynaptic membrane (*p* = 0.0005, Mann–Whitney test). ***D***, Percentage of the total dendritic spine gold particles that localized directly at the PSD or perisynaptic regions within 200 nm of the PSD (*n* = 27 gold particles). ***E***, Representative image of a dendritic segment processed for ExM, labeled for presynaptic bassoon (green), postsynaptic GluA1 (red), and Aβo (cyan). The lower panels show two representative synapses (labeled 1 and 2) from the larger image and their respective 3D volume renderings. ***F***, top, Schematic of the analysis used to quantify presynaptic and postsynaptic Aβo signal. The synaptic axis is defined by a line drawn between the centers of mass of masked bassoon (presynaptic marker) and GluA1 (postsynaptic marker) signals. A vector is generated from the middle of the pre/post axis to the center of mass of the segmented Aβo, with the component vectors representing the radial distance from the synapse center (r) and the distance along the pre/post axis (*z*). Bottom, Quantification of Aβo signal along the pre/post and radial axes. Negative and positive values indicate postsynaptic and presynaptic localization respectively. The number of Aβo puncta at different distances along the pre/post axis are summed and plotted in the histogram to the right (*n* = 89 synapses from 26 neurons from 3 independent cultures).

Aβo is reported to rapidly disrupt both presynaptic and postsynaptic function, yet the relative extent of direct Aβo association with axonal terminals and dendritic spines remains largely uncharacterized. To address this, we first performed postembedding immunogold EM. We treated hippocampal cultures for 10–45 min with Aβo (500 nm) or an equal volume of PBS (negative control) before fixation. Following fixation, embedding, and cutting, we labeled sections with an Aβo antibody and a gold-conjugated secondary antibody. We imaged samples by scanning EM and quantified the number of gold particles per linear micrometer of presynaptic or postsynaptic membrane (Fig. 4*A*). Given the estimated size of the primary and secondary antibodies (∼10 nm each), we only included gold particles that were within 20 nm of the plasma membrane. Importantly, we observed little background signal in control (no added Aβo) samples (Fig. 4*B*). In samples treated with Aβo, 32.5% and 67.5% of the total synaptic signal was observed at the presynaptic and postsynaptic membrane respectively (Fig. 4*C*). Of the total postsynaptic signal, ∼75% was nonoverlapping with the PSD, consistent with our dSTORM and SIM data demonstrating perisynaptic binding (Figs. 3, 4*D*).

Given the sparse labeling and extensive fixation/processing steps required for immunogold-EM, we took an independent fluorescence-based approach to quantify presynaptic and postsynaptic Aβo. We used ExM, which allows acquisition of three or more fluorescent labels (an advantage over dSTORM) with higher spatial resolution compared with SIM. We labeled live hippocampal neurons with Aβo and then fixed and stained for bassoon (presynaptic), GluA1 (postsynaptic), and Aβ and expanded the preparation according to (Zhang et al., 2020; Fig. 4*E*). Using cellular nuclei as a reference, we estimate our samples were expanded 4-fold. We quantified Aβo signal in 3D with respect to a plane perpendicular to an axis connecting the center of mass of the segmented presynaptic and postsynaptic signals (Fig. 4*F*; for details, see Materials and Methods). We found that 40.8% and 59.2% of Aβo signal associated with the presynaptic and postsynaptic label respectively, in agreement with our immunogold labeling. Once more, Aβo did not directly overlap with synaptic markers (Fig. 4*E*,*F*). Combined, these experiments pinpoint Aβo binding to sites immediately adjacent to the synaptic cleft with significant amounts of Aβo directly binding both presynaptic and postsynaptic compartments.

### Plasticity is disrupted specifically at Aβo-bound spines

**Figure 5.**
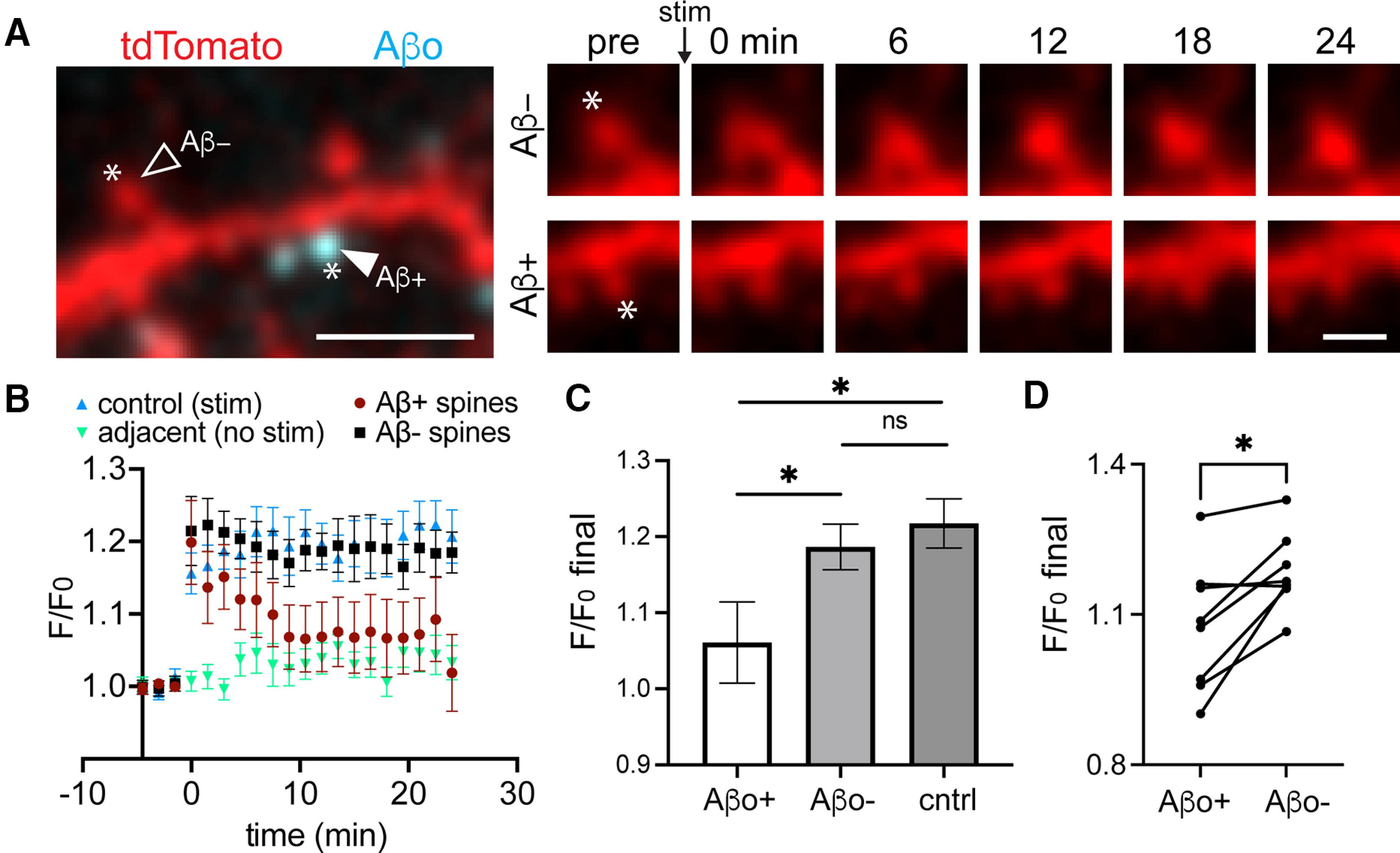
Aβo-mediated plasticity impairment is locally restricted near sites of surface binding. ***A***, left, Representative image of a dendritic segment from a neuron transfected with tdTomato (red) and treated with Aβo-488 (teal). Asterisks designate the location of MNI-glutamate uncaging. The closed arrowhead shows a spine with bound Aβo (Aβ+), and the open arrowhead shows a neighboring spine lacking Aβo (Aβ–). Right, Time course of Aβo– spine (top) and Aβo+ spine (bottom) from the same dendritic segment before the uncaging stimulus and up to 24 min following the stimulus. Scale bars: 5 μm (left panel) and 1 μm (right panels). ***B***, Quantification of spine size (based on the cell fill intensity) before and after MNI-glutamate uncaging for control spines not treated with Aβo (blue, *n* = 14 spines, *N* = 5 neurons, 3 independent cultures), adjacent spines that were not stimulated (green, *n* = 12 spines, *N* = 5 neurons, 3 independent cultures), Aβ+ spines from cultures treated with 500 nm Aβo for at least 25 min (maroon, *n* = 16 spines, *N* = 8 neurons, 3 independent cultures), and neighboring Aβo-lacking spines (black, *n* = 14 spines, 8 neurons, 3 independent cultures). ***C***, Average increase in spine cell fill signal during the final 3 min of imaging compared with baseline for control (*n* = 14 spines, *N* = 5 neurons, 3 independent cultures), Aβo+ (*n* = 16 spines, *N* = 8 neurons, 3 independent cultures), and Aβo– (*n* = 14 spines, 8 neurons, 3 independent cultures; *p ≤ 0.05, Student’s *t* test). ns = not significant. ***D***, Average F/F_0_ over the final 3 min of imaging compared with baseline at Aβo-bound spines and neighboring Aβo-free spines on the same neurons (eight neurons, three independent cultures, *p = 0.0116, paired *t* test).

While most synapses associate with Aβo at the concentration used here (500 nm), a small fraction of spines do not appear to bind Aβo. This allowed us to test whether Aβo selectively impairs synapses to which it is bound (Fig. 5*A*). One of the hallmark synaptic pathologies of Aβo is LTP impairment. LTP is associated with structural enlargement of dendritic spines (sLTP) and can be locally triggered at targeted synapses using focal two-photon glutamate uncaging (Matsuzaki et al., 2004). We used an established uncaging protocol (45 pulses, 0.5 Hz) to induce sLTP at individual Aβo-bound and Aβo-free spines (Lee et al., 2009). In control cells that were not treated with Aβo, our glutamate uncaging protocol triggered robust and persistent spine growth (Fig. 5*B*,*C*). Aβo-bound spines from cells treated with Aβo for 30–45 min before sLTP induction initially exhibited a comparable degree of growth as controls, but returned to baseline levels several minutes following plasticity induction. Surprisingly, neighboring Aβo-free spines exhibited persistent growth that was indistinguishable from control spines. In nearly every case, Aβo-free spines exhibited increased persistent growth compared with nearby Aβo-bound spines on the same cell (Fig. 5*D*). Since the extent of spine growth depends on initial size, we compared the average baseline sizes (calculated as spine area from 2D-projected images) of Aβo-bound and unbound spines targeted for sLTP. There was no significant difference between the two populations (Aβo-bound, 1.76 ± 0.25 μm^2^; Aβo-free 1.69 ± 0.19 μm^2^). Thus, Aβo disrupts plasticity in a spatially restricted manner, presumably through local signaling mechanisms constrained near sites of Aβo surface engagement.

## Discussion

Increasing evidence supports a role for secreted, soluble Aβo as the primary culprit in AD-associated synapse dysfunction. While many studies have characterized steady state Aβ distribution in human postmortem samples and animal models, little is known about the earliest steps of Aβo-mediated synapse toxicity, including the spatial and temporal dynamics of its engagement with the neuronal membrane. Here, we used a combination of live and high-resolution imaging modalities to define the rate, stability, nanoscale localization and functional consequences of Aβo binding.

To our knowledge, ours is the first to directly visualize Aβo longitudinally as it associates with neurons. This analysis revealed that Aβo generally nucleates at specific sites and attracts additional Aβo with cluster growth saturating within minutes rather than binding as discrete, preformed assemblies. Growth could occur through recruitment of additional Aβo from soluble pools and/or by coalescence of laterally diffusing Aβo/receptor complexes on the cell surface (Renner et al., 2010). In either case, the rapid surface assembly and synaptic association we observe is consistent with numerous observations that Aβo can rapidly (within minutes) affect synaptic function (Shankar et al., 2008; Laurén et al., 2009; Um et al., 2012; Freund et al., 2016; Cook et al., 2019; Gulisano et al., 2019). It is important to note that we observed little Aβo internalization over this timescale, consistent with action through a signaling surface receptor(s) rather than direct Aβo-mediated interference of intracellular plasticity-related processes. However, our data do not rule out an intracellular role for Aβo in pathologies that manifest over longer timescales, such as synapse elimination or cell death (Takahashi et al., 2002, 2004). Indeed, numerous studies have demonstrated accumulated intracellular pools of Aβ at synaptic sites in animal models and human AD samples where Aβ is constitutively produced for months or years (Koffie et al., 2009; Pickett et al., 2016).

While we focused on Aβo binding at synaptic sites, it should be noted that Aβo accumulates at synaptic and nonsynaptic sites with indistinguishable kinetics, suggesting widespread and heterogeneous distribution of Aβo receptor(s). The rate of Aβo binding was similar at spines and nonsynaptic sites but surface Aβo clusters were consistently more intense near excitatory synapses suggesting some degree of receptor enrichment at synaptic sites. While intriguing, the functional significance of synaptic Aβo binding has remained unclear. We demonstrate for the first time that Aβo-bound spines are more susceptible to plasticity disruption than neighboring Aβo-free spines, supporting a model where Aβo engages locally restricted signaling mechanisms to impair plasticity. Our experiments were performed over a relatively short timescale, with application of Aβo ∼30 min before plasticity induction. It remains possible that longer, disease relevant exposure times would lead to more global plasticity disruption. It will also be important to investigate whether other aspects of Aβo-mediated pathology, such as synapse loss, occur selectively at sites where Aβo initially engages the neuronal surface. In any case, these results emphasize the importance of future experiments unraveling molecular features that shield select synapses from Aβo binding and subsequent plasticity deficits.

Our experiments are also the first to map the nanoscale distribution of Aβo surface engagement. The imaging techniques used in earlier studies lacked the spatial resolution to precisely map Aβo binding sites (Lacor et al., 2004; Koffie et al., 2009; Renner et al., 2010; Um et al., 2012). Surprisingly, we observed very little Aβo actually binds directly at the postsynaptic density. Instead, Aβo forms nanoscale clusters immediately adjacent to and surrounding the excitatory synaptic cleft. Several reported Aβo receptors localize to perisynaptic regions, including α7 nicotinic acetylcholine receptor (α7-nAchR), cellular prion protein (PrP^c^), and metabotropic glutamate receptor 5 (mGluR5; Luján et al., 1996; Mironov et al., 2003; Jones and Wonnacott, 2004). Perisynaptic localization also suggests Aβo is unlikely to exert its effects through direct binding of synaptic neurotransmitter receptors such as NMDA or AMPA-type glutamate receptors as previously proposed (De Felice et al., 2007; Lacor et al., 2007; Zhao et al., 2010; Texidó et al., 2011). While earlier studies concluded Aβo binds primarily to postsynaptic sites on dendritic spines, these experiments primarily relied on diffraction limited imaging techniques. Using multiple approaches, we confirmed binding near the postsynaptic membrane but also observed a substantial fraction of Aβo binding to axonal terminals, consistent with rapid Aβo-mediated effects on presynaptic vesicle release probability, glutamate reuptake and structural alterations (Abramov et al., 2009; Huang et al., 2013; He et al., 2019). Whether the same receptor mediates Aβo binding to both presynaptic and postsynaptic compartments is unknown, but several reported Aβo receptors localize to both sides of the synapse, including α7-nAchR and PrP^c^ (Moya et al., 2000; Fabian-Fine et al., 2001; Barmada et al., 2004; Um et al., 2012, 2013).

Taken together, our study is the first to interrogate the kinetics, stability, ultrastructural localization and functional consequences of Aβo binding. These basic, yet fundamental assessments provide new insight into the earliest steps of Aβo toxicity and lay the groundwork for future studies evaluating the relevant receptor(s) responsible for neuronal surface engagement and the local signaling mechanisms leading to synapse dysfunction.

## Synthesis

Reviewing Editor: Karl Herrup, University of Pittsburgh School of Medicine Decisions are customarily a result of the Reviewing Editor and the peer reviewers coming together and discussing their recommendations until a consensus is reached. When revisions are invited, a fact-based synthesis statement explaining their decision and outlining what is needed to prepare a revision will be listed below. The following reviewer(s) agreed to reveal their identity: Emmanuel Prikas. Note: If this manuscript was transferred from JNeurosci and a decision was made to accept the manuscript without peer review, a brief statement to this effect will instead be what is listed below.

This is an interesting manuscript which provides high resolution longitudinal imaging of Aβ olilgomer accumulation at the synapses in neuronal cultures and its functional effects on synaptic function. The manuscript is beautifully illustrated with high quality images. The conclusions are well supported by the experimental data. The work stands out for its use of complimentary, high resolution imaging techniques to show amyloid beta oligomers (A-betaO) as they associate - and distribute - on extra-synaptic surface. the authors extend the study to the functional realm by showing that A-betaO-bound spines is correlated with reduced LTP-dependent structural changes.

The consensus of the reviewers and the Editor is that this is a very strong manuscript that will be a welcome contribution to the pages of eNeuro with only a few minor issues needing some additional work before moving to publication.

There was a sense that the concept of “synapse” was not clearly defined. For example, in the Abstract, the text reads, “Surprisingly, Abo does not bind directly at synapses as previously thought, but instead forms distinct nanoscale clusters encircling the postsynaptic membrane with a significant fraction also binding presynaptic terminals.” The feeling was that the language here could be much more precise. The synapse is a structure that includes the axon terminal and the postsynaptic dendritic spines. The meaning of the statement that “Abo does not bind directly at synapses as previously thought” is clear, but it might be better if the morphological terms were better defined.

Similarly in the main text conclusion the text reads “Abo does not bind directly at synapses, but instead forms stable nanoscale clusters encircling the postsynaptic membrane with a significant fraction also binding the presynaptic terminal.” Again, the meaning is clear, but the reviewers felt that it would be more accurate to rephrase this sentence since the presynaptic terminal is part of the synapse. Indeed, the postsynaptic membrane (dendritic spines) is also part of the synapse. There is no need for massive changes only small tweaks of language.

One other minor suggestion that the authors might wish to consider is that the spatial distribution of A-betaO at pre- and post-synapses, but not PSDs, raises the question of what might be the common features leading to A-betaO binding in these regions. Some discussion of this question might be included.

Congratulations on a work well done.

## Author Response

Dear Dr. Herrup,

We would like to thank you and the reviewers for the rapid and thoughtful consensus review of our manuscript “Precision mapping of amyloid beta binding reveals perisynaptic localization and spatially restricted plasticity deficits” (eN-TNWR-0416-21X). We have addressed the minor issues raised by textual changes to the revised manuscript as suggested by the reviewers (please see point by point response below for details).

Point by point response to the consensus review:

The consensus of the reviewers and the Editor is that this is a very strong manuscript that will be a welcome contribution to the pages of eNeuro with only a few minor issues needing some additional work before moving to publication.

Response: We thank the reviewers for the positive feedback on our work.

There was a sense that the concept of “synapse” was not clearly defined. For example, in the Abstract, the text reads, “Surprisingly, Abo does not bind directly at synapses as previously thought, but instead forms distinct nanoscale clusters encircling the postsynaptic membrane with a significant fraction also binding presynaptic terminals.” The feeling was that the language here could be much more precise. The synapse is a structure that includes the axon terminal and the postsynaptic dendritic spines. The meaning of the statement that “Abo does not bind directly at synapses as previously thought” is clear, but it might be better if the morphological terms were better defined.

Response: This is an excellent suggestion and we have rephrased the referenced statements accordingly throughout the manuscript. Specifically, we have changed “synapse” to “synaptic cleft” to clarify this observation. We think this should avoid any further confusion pertaining to the nanoscale localization of Abeta at perisynaptic sites. Changes are highlighted in bold in the revised manuscript (see pp. 2, 4, 24, 26, 30)

Similarly in the main text conclusion the text reads “Abo does not bind directly at synapses, but instead forms stable nanoscale clusters encircling the postsynaptic membrane with a significant fraction also binding the presynaptic terminal.” Again, the meaning is clear, but the reviewers felt that it would be more accurate to rephrase this sentence since the presynaptic terminal is part of the synapse. Indeed, the postsynaptic membrane (dendritic spines) is also part of the synapse. There is no need for massive changes only small tweaks of language.

Response: Please see response to the first point above.

One other minor suggestion that the authors might wish to consider is that the spatial distribution of A-betaO at pre- and post-synapses, but not PSDs, raises the question of what might be the common features leading to A-betaO binding in these regions. Some discussion of this question might be included.

Response: In the discussion section of the original manuscript, we highlighted putative receptor proteins that share a similar localization pattern to that observed for Abeta (i.e. perisynaptic, located both pre- and postsynatpically). This discussion is on pg. 30 of the revised manuscript.
