# Supplementary figures and images for: Precision Mapping of Amyloid-β Binding Reveals Perisynaptic Localization and Spatially Restricted Plasticity Deficits
Source: eNeuro. 2021 Dec 10;8(6):ENEURO.0416-21.2021. doi: 10.1523/ENEURO.0416-21.2021 (PMC8687484; doi:10.1523/ENEURO.0416-21.2021)

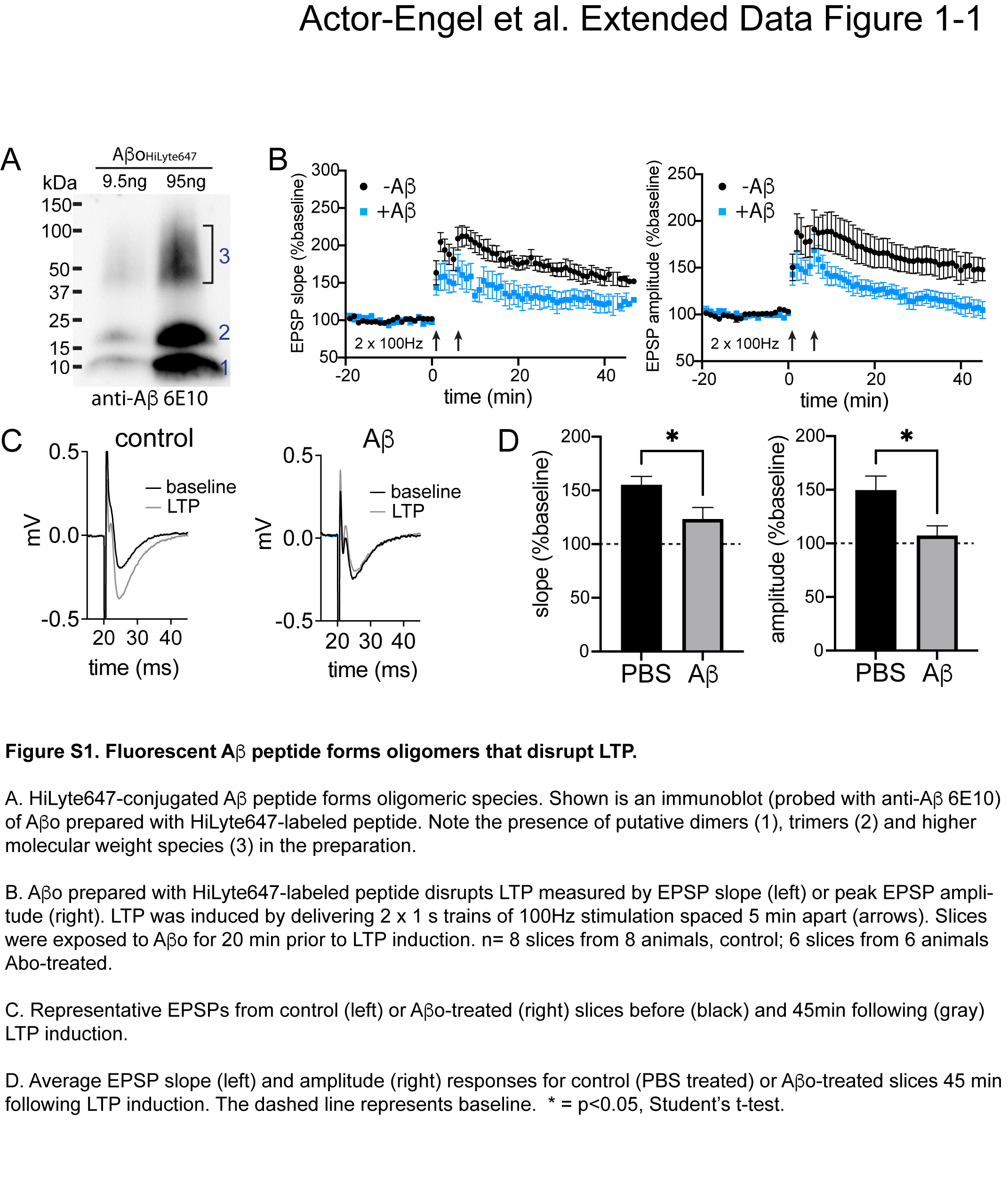

Supplement: Extended Data Figure 1-1 — Fluorescent Aβ peptide forms oligomers that disrupt LTP. A, HiLyte647-conjugated Aβ peptide forms oligomeric species. Shown is an immunoblot (probed with anti-Aβ 6E10) of Aβo prepared with HiLyte647-labeled peptide. Note the presence of putative dimers (1), trimers (2), and higher molecular weight species (3) in the preparation. B, Aβo prepared with HiLyte647-labeled peptide disrupts LTP measured by EPSP slope (left) or peak EPSP amplitude (right). LTP was induced by delivering 2 × 1-s trains of 100-Hz stimulation spaced 5 min apart (arrows). Slices were exposed to Aβo for 20 min prior to LTP induction. n = 8 slices from 8 animals, control; 6 slices from 6 animals Aβo treated. C, Representative EPSPs from control (left) or Aβo-treated (right) slices before (black) and 45 min following (gray) LTP induction. D, Average EPSP slope (left) and amplitude (right) for control (PBS treated) or Aβo-treated slices 45 min following LTP induction. The dashed line represents baseline; *p < 0.05, Student’s t test. Download Figure 1-1, TIF file. [file enu-eN-NWR-0416-21-s01.tif]
